# Methods of increasing cultural competence in nurses working in clinical practice: A scoping review of literature 2011–2021

**DOI:** 10.3389/fpsyg.2022.936181

**Published:** 2022-08-24

**Authors:** Martin Červený, Inka Kratochvílová, Věra Hellerová, Valérie Tóthová

**Affiliations:** Faculty of Health and Social Sciences, Institute of Nursing, Midwifery and Emergency Care, University of South Bohemia, České Budějovice, Czechia

**Keywords:** education, training, cultural competence, nursing, development

## Abstract

**Aim:**

Training for the development of cultural competence is often not part of the professional training of nurses within the European Economic Area. Demographic changes in society and the cultural diversity of patients require nurses and other medical staff to provide the highest quality healthcare to patients from different cultural backgrounds. Therefore, nurses must acquire the necessary cultural knowledge, skills, and attitudes as part of their training and professional development to provide culturally competent care to achieve this objective.

**Objective:**

This review aims to summarize existing methods of developing cultural competence in nurses working in clinical practice.

**Design:**

A scoping review of the literature.

**Method:**

The following databases were used: PubMed, ScienceDirect, ERIH Plus, and Web of Science using keywords; study dates were from 2011 to 2021.

**Results:**

The analysis included six studies that met the selection criteria. The studies were categorized as face-to-face, simulations, and online education learning methods.

**Conclusion:**

Educational training for cultural competence is necessary for today’s nursing. The training content should include real examples from practice, additional time for self-study using modules, and an assessment of personal attitudes toward cultural differences.

## Introduction

Current demographic changes mean that nurses need to provide quality nursing care for patients from different cultural backgrounds. Horvat et al. (2014) report that health workers will increasingly be obliged to provide healthcare to patients from different cultural groups. Eurostat (2019) states that 4.2 million people from other countries migrated to the European Union in 2019. Germany (88,630), France (29,910), Spain (29,620), and Romania (23,370) reported the largest number of immigrants. Cruz et al. (2017a) draw attention to the fact that every population group has unique norms, values, and practices that determine the group’s perception of health, which is why it is important to implement the principles of culturally specific healthcare.

### Cultural competence in nursing

Cultural competence (Ahn, 2017) in nursing care is essential for providing quality care for patients from different cultural backgrounds. It is a specific concept related to transcultural nursing and contains a wealth of skills and knowledge regarding cultural values, health beliefs, religion, and human philosophy. It is a concept linked to culturally specific nursing care (Leininger and McFarland, 2002). Cultural competence in nursing has been defined as a set of knowledge, skills, and attitudes applied in the clinical practice of nursing in an intercultural context (Cerezo et al., 2014; Paric et al., 2021).

### Development of cultural competence of nurses

According to Horvat et al. (2014), the development of cultural competencies is a crucial component for addressing health disparities and strategies to improve culturally competent care, and many experts agree (Harkess and Kaddoura, 2016; Mariño et al., 2018; Curtis et al., 2019; Červený et al., 2020; Swihart et al., 2021). Faber (2021) adds that the education of health professionals is also a method of addressing racial and ethnic discrimination resulting from structural inequality. According to Carey (2011), nursing schools should provide adequate opportunities to develop cultural competence. Cruz et al. (2017b) recommend that nursing schools include international standards for culturally competent nursing care.

Moreover, teaching standards should be adapted to local cultural diversity within each country. This ensures that nurses have a proper cultural context that can promote the development of cultural sensitivity, cultural adaptability, and cultural motivation. This type of education is demanding for teachers, who need to have the most up-to-date information from professional literature, constantly evaluate self-esteem, and modify educational methods to develop cultural competence (Prosen and Bošković, 2020). However, according to Faber (2021), there is a wealth of evidence in literature where researchers present the effectiveness of cultural competence training in individual health professions to be more linguistically and culturally aware. Farber also states that there are no coherent sector-wide standards for defining cultural competence, educational practice, evaluation measures, or target results.

### Why is a literature review essential?

Accelerating globalization and demographic changes in society, the incidence of patients from different cultural backgrounds, language barriers, discrimination, racism, prejudice, and stereotypes are all factors that affect the quality of nursing care (Červený et al., in press; Shepherd et al., 2019; Williams et al., 2019; Joo and Liu, 2020). Prosen (2018) states that providing culturally competent nursing care for patients from different cultural backgrounds should not be seen as a privilege but as a human right. In order to eliminate barriers to quality care, it is necessary to find the best possible methods for developing cultural competence in nurses in clinical practice.

### Research question

1. What methods are effective at increasing the level of cultural competence?
2. What factors can improve existing methods of increasing the level of cultural competence?

### Aim of literature overview

The main objective of the review was to summarize the existing methods of developing cultural competence in nurses working in clinical practice.

1. Determine which educational methods effectively increase cultural competence in clinical practice.
2. Identify the impact of education on cultural competence.
3. Identify potential opportunities to improve the development of cultural competence.

## Materials and methods

**Figure 1:**
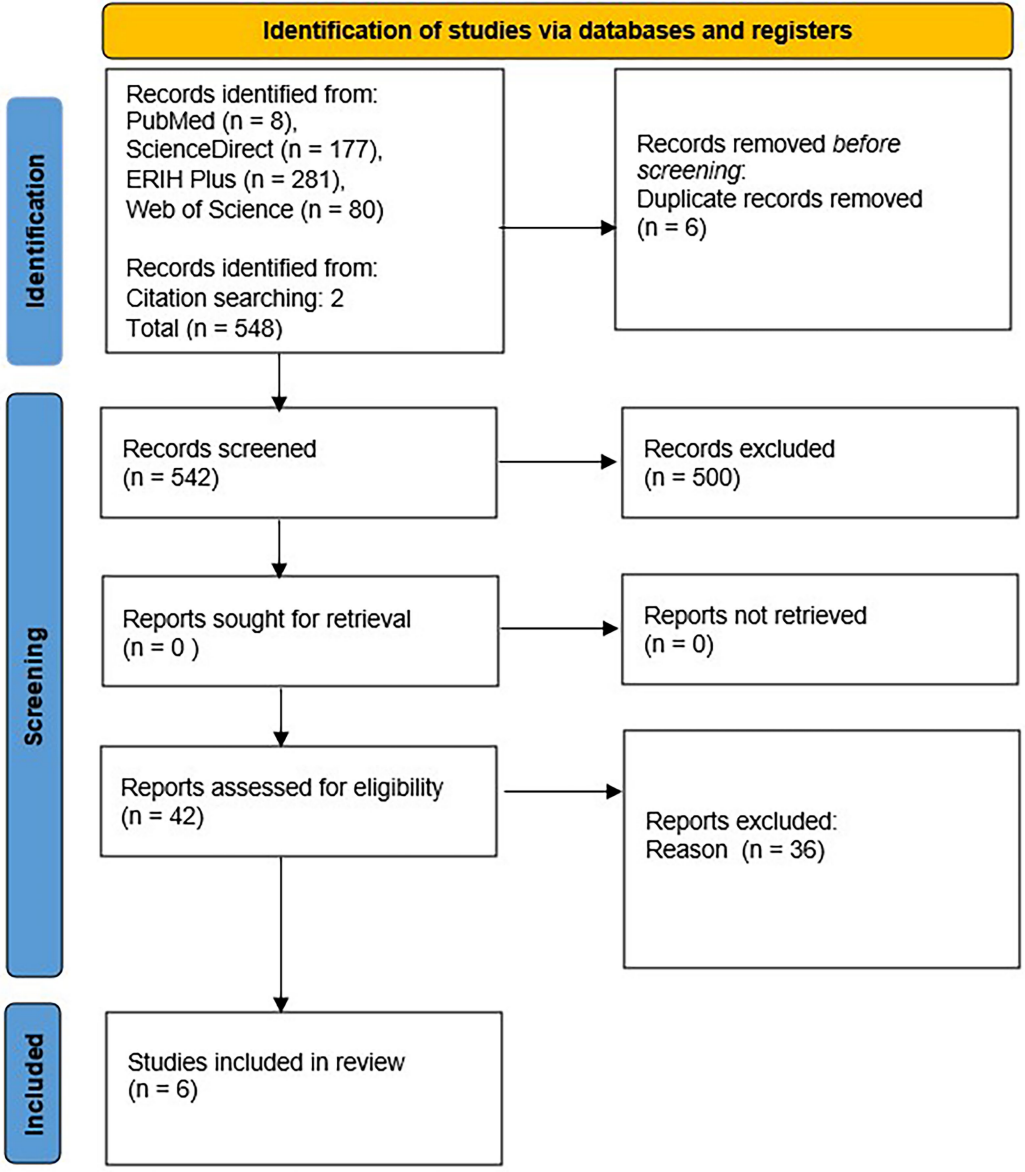
PRISMA flow diagram of the scoping review.

This study is based on a qualitative scoping review using the Preferred Reporting Items for Systematic Reviews and a Meta-Analyses Extension for Scoping review ([PRISMA-ScR], Tricco et al., 2018; Page et al., 2021; Figure 1) and the Participants, Interventions, Comparison, and Outcomes (PICO) listed in Table 1.

**Table 1:** Inclusion and Exclusion criteria for the scoping review.

Inclusion criteria	Exclusion criteria
Nurses or healthcare professionals working in clinical practice	Nursing students
Published in English	Retired nurses not providing direct patient care
Published from 2011 to 2021	Not original research: opinion, editorial, conference abstract, systematic reviews
Qualitative and quantitative studies	Articles not available in English

### Methods of searching the literature

The analyzed publications were collected from the PubMed, ScienceDirect, ERIH Plus, and Web of Science databases using keywords and Booleans operatives: (“transcultural education”) OR (“training”) AND (“culturally competence”) AND (“nurses”) AND (“clinical practice”). All sources were academic publications that went through the peer-review process. The focus of this review was on the following elements:

- Population: Clinical practice nurses
- Intervention: Education to increase cultural competences
- Related: Clinical practice nurses
- Outcome: Increasing cultural competencies in clinical practice nurses through education (training)

The criteria for the selection of resources are presented in Table 1. We searched for resources dated from 01.12.2011 to 31.12.2021.

### Data charting, extraction, and quality evaluation

We used a 3-step screening process that was evaluated in MS Excel. In the first step, we searched the article’s title and abstract. In the second step, we identified and sorted articles that met the outline ranking criteria and assessed their quality. To evaluate the articles’ quality, two co-authors independently used the Critical Appraisal Skills Programe (2018). This general tool evaluates any qualitative methodology. It has 10 questions asking the researcher to assess whether appropriate research methods were used and whether the findings were presented meaningfully (Červený et al., 2020; Long et al., 2020). The results of the quality assessment are presented in Table 2. In the third step, the data were extracted.

**Table 2:** Results of critical appraisal checklist results. Questions of quality, author(s), year, country

	Celik et al., 2012, NLD	Perry et al., 2015, AUS	Ahn, 2017, ROK	Kaihlanen et al., 2019, FIN	Slobodin et al., 2021, ISR	McDonald et al., 2021, SWE
1. Was there a clear statement of the aims of the research?	Y	N	Y	Y	Y	Y
2. Is the qualitative methodology appropriate?	Y	N^1^/CT^2^	N	Y	Y^1^/CT^2^	Y
3. Was the research design appropriate to address the aims of the research?	Y	CT^1^/Y^2^	Y	Y	Y	Y
4. Was the recruitment strategy appropriate to the aims of the research?	Y	Y	Y	Y	Y	Y
5. Was the data collected in a way that addressed the research issue?	Y	Y	Y	Y^1^/N^2^	Y	Y
6. Has the relationship between researcher and participants been adequately considered?	Y	Y	Y	Y	Y	Y
7. Have ethical issues been taken into consideration?	Y	Y	Y	Y	N^1^/Y^2^	Y
8. Was the data analysis sufficiently rigorous?	Y	N	Y	Y^1^/N^2^	Y	Y
9. Is there a clear statement of findings?	Y	Y	Y	Y	Y	Y
10. How valuable is the research?	Y	Y	Y	N^1^/CT^2^	Y	Y
Final quality level/grade						

Y, Yes; N, No; CT, Cannot tell; ROK, Republic of South Korea; ISR, Israel; SWE, Sweden; AUS, Australia; NDL, Netherlands (the); FIN, Finland.

1 Co-authors answers-by IK.

2 Co-authors answers-by VH.

A total of 548 articles were identified based on database searches, and two other articles were added to the analysis because they met the criteria for selecting articles. After removing duplicates, we approached the analysis of titles and abstracts of individual articles. Based on the analyses of abstracts, we discarded 500 articles. Forty-two articles were selected for full-text analysis, but we discarded another 36 articles after analysis. The articles included in the scoping review were re-analyzed a week after the first reading to avoid erroneous conclusions. The data were sorted, encoded, and categorized into three themes: (1) *Methods of increasing cultural competence, (2) The impact of education on the cultural competence of participants, and (3) Possibilities for developing educational programs in the field of cultural competence.*

## Results

### Characteristics of articles

The articles included in the analysis were published from 2011 to 2021. The articles came from 6 countries: South Korea (Ahn, 2017), Israel (Slobodin et al., 2021), Sweden (McDonald et al., 2021), Australia (Perry et al., 2015), the Netherlands (Celik et al., 2012), and Finland (Kaihlanen et al., 2019). Three articles used a mixed-method method (Celik et al., 2012; Perry et al., 2015; McDonald et al., 2021). One article was based on a cross-sectional study (Ahn, 2017), and one article used an online education intervention study (Slobodin et al., 2021). Only one article utilized a qualitative study (Kaihlanen et al., 2019).

In terms of study participants, in the study by Celik et al. (2012), there were 31 paramedics, two psychiatric hospital nurses, six hospital nurses, and four nursing home nurses. Kaihlanen et al. (2019) included 20 nurses in their training program. Nurses were explicitly included in all analyzed articles, except for the study by Slobodin et al. (2021), in which participants were described as healthcare professionals, but no further details were provided. Table 3 provides an overview of the studies included in this scoping review.

**Table 3:** Characteristics of included studies.

Author(s), Year	Participants	Methods	Content of training to increase cultural competence
Kaihlanen et al., 2019	Registered nurses (*n* = 14); practical nurses (*n* = 6)	Qualitative study	16-h Face to Face training. The training was based on sociocultural differences, perception of pain in individual cultures, personality differences, knowledge from various cultural experts, and knowledge gained from self-reflection.
Slobodin et al., 2021	Healthcare professionals (*n* = 303)	Pre-post web-based intervention study	An online educational program from the historical perspective of the pandemic; program objectives evaluated cultural challenges in the health sector, the importance of cultural competence in emergencies, cultural competence, knowledge, and skills in the context of COVID-19.
Celik et al., 2012	Healthcare professionals (*n* = 31)	Mix-method Quantitative and Qualitative methods	The training program was based on the Deming cycle and was divided into four modules. The training focused on conceptualizing differences in healthcare between the healthcare professionals and applying instructions to address diversity in practice.
Perry et al., 2015	Healthcare professionals (*n* = 60)	Mix-method Quantitative and Qualitative methods	eSimulation module was based on developing participants’ knowledge and skills to understand the role of language in healthcare and highlighting the benefits of using an interpreter in clinical work. The use of open-ended, culturally sensitive issues to address language and cultural problems at patient discharge.
Ahn, 2017	Nurses (*n* = 275)	Cross-sectional design and structured equation modeling	Hypothetical model for the development of cultural competence.
McDonald et al., 2021	Mental Healthcare professionals (*n* = 248)	Mix-method evaluation	Comprehensive Cross-Cultural Training included interactive lectures on cultures, psychopathology, migration discussions, and refugee-related studies.

Theme 1: Methods of increasing cultural competence

Methods for developing cultural competencies in nurses are presented in Table 3.

An online educational program was used in the study by Perry et al. (2015) and Slobodin et al. (2021). Slobodin et al. (2021) divided their training sessions into eight modules lasting about 30 min. Their training was linked to the pandemic situation; therefore, the online training course included a historical review of the pandemic and its impact on the social fabric of society. The study by Perry et al. (2015) included modules lasting about 60 min that focused on understanding the importance of language in the healthcare environment, using interpreters in clinical practice, and addressing linguistic and cultural issues during patient discharge from the hospital. Celik et al. (2012) used a modified six-phase Deming cycle during four training sessions. As the authors stated, the first phase was an attention-free phase (Unawareness), where health professionals were unaware of diversity factors in healthcare and thought these factors or questions were irrelevant to clinical practice. The second phase was the phase of ‘limited” awareness, where healthcare workers realize that diversity factors exist but do not implement them in clinical practice. The first two phases, which the authors added, were followed by the usual phases of the Deming cycle (Plan, Do, Study or Check, and Act). The (Plan) in their study means: deliberately paying attention to diversity in clinical practice, the (Do) means to implement knowledge into clinical practice, the (Study or Check) means evaluating the results after implementation of culturally diverse care, and the (Act) means the implementation of modified nursing care based on that process.

McDonald et al. (2021) used Comprehensive Cross-Cultural Training (CCCT), developed in 2016, in response to a health crisis. The authors carried out 12 all-day training and two half-day interventions in this study. In the Kaihlanen et al. (2019) study, training included 16 h of full-time teaching, divided into four, 4-h modules, which ran once a week for 4 weeks. The training timing encouraged trainees to implement the acquired knowledge into practice quickly. The first training focused on the issue of culture (What is culture), the second training involved awareness of one’s own culture (Culture in me), the third training covered communication, and the last training focused on understanding attitudes (Meaning of conviction). The teaching method was “storytelling,” where the lecturer used real-life experiences from practice and images to demonstrate the cultural aspects of diversity. The image presentation was intended to make participants realize that people with different cultural backgrounds perceive the same image differently. After each module, there was a discussion to assess cultural features and understand why it is essential to apply culturally specific facts to the care of patients. Participants were given access to a Web-based learning platform where they could anonymously share their thoughts with others.

Theme 2: Impact of cultural competence education on participants

Kaihlanen et al. (2019) used three semi-structural small-group interviews, which focused on the general usefulness of training, personal usefulness, usefulness for patients, quality of training, and suggestions for improvement. The participants in the training welcomed the fact that the training was not entirely focused only on cultural competence in healthcare. The lecturer was not a healthcare professional and integrated new ideas and insights into actual clinical practice in the hospital. A positive impact can be seen as a general and open debate on cultural issues, which are often not part of the general working culture. Small training groups also facilitated participant involvement in the discussion. After completing the training, participants felt more open-minded and focused on caring for patients with different cultural backgrounds. The training also drew the attention of participants to inappropriate communication skills. The training also benefited patients since participants exited the training with better attitudes, awareness, and ability to recognize and respect the cultural background of the patient without imposing stereotypes and prejudices. After completing the training, most participants stated that they no longer had to use checklists or guidance for treating patients from different cultural backgrounds; however, they continued to express uncertainty regarding religious issues.

Celik et al. (2012), McDonald et al. (2021), and Slobodin et al. (2021) used pre and post-tests to determine the effect of individual training on cultural competence. Perry et al. (2015) used only post-testing. The post-test used by McDonald et al. (2021) statistically confirmed that participants who had experience with patients from different cultural backgrounds had higher cultural assessments than participants who did not. A similar relationship was seen regarding the use of interpreting services. The study Focus-Group showed that CCCT training significantly contributed to a better understanding of cultural competence, cultural viewpoints, and cultural phrases in patients from different cultural backgrounds.

The trainees received important information about migration and being an immigrant and understood that they needed to act to benefit the patient (McDonald et al., 2021). The use of eSimulations also significantly impacted the cultural awareness of graduates (Perry et al., 2015). After completing eSimulation training, post-survey questionnaires reported better communication and a better understanding of language and culture in the context of healthcare, as well as the benefits of using an interpreter when talking to patients from different cultural backgrounds. Participants also expressed new awareness of their assumptions about patients and the dangers of hasty conclusions involving cultural issues in patient care and planning. An online training study by Slobodin et al. (2021) found that only two independent variables had a statistically significant impact on cultural competence (1) the pre-intervention level of self-reported cultural competence (*p* = 0.005) and (2) exposure to previous cultural competence training as part of their overall educational framework. After completing training, the most significant gains were seen relative to culturally competent attitudes, meetings, and skills, and the smallest gains were seen in overall knowledge.

Celik et al. (2012) reported that the degree of cultural awareness improved significantly in mental health workers (*p* = 0.026) and hospital workers (*p* < 0.005). Improvements for those working in nursing homes were not statistically significant (*p* = 0.749). Participants said they became more critical of a neutral approach to diversity and had not previously considered diversity relevant to healthcare, although they reported that they better perceived each patient as unique, with each having specific health needs.

Theme 3: Opportunities for the development of cultural competence education

The research by Ahn (2017) used a questionnaire investigation to verify the hypothetical model of cultural competence in nurses. The following measuring tools were used: Multicultural Experiences Questionnaire, a Generalized Ethnocentrism Scale, a Cultural Competence Assessment Instrument, the Low and High Context Measure of Attributional Confidence Scale, the Intergroup Anxiety Scale, the Cybernetic Coping Scale, and the Cultural Competence Scale for Clinical Nurses. The authors found that multicultural experience, ethnocentric attitudes, organizational competence support, intercultural anxiety, and coping strategies have statistically significant direct and indirect impacts on cultural competence.

Coping strategies were seen to have a direct impact on cultural competence. Kaihlanen et al. (2019) examined methods for developing cultural competence training. They suggested using real examples, open discussion, and the lecturer’s expertise. However, training participants noted that trainers with other cultural backgrounds should also be included. Trainees suggested that (1) materials should be available online, (2) training should take less time, and (3) each training should be followed by a one-week break (participants said they felt time pressure to complete the assigned tasks). Additionally, more time between training would allow time for reflection on training content. Participants in the study by Celik et al. (2012) also suggested that there be more time between training sessions, again to provide more time to reflect on the concepts of cultural diversity.

## Discussion

This scoping review summarizes the available resources on developing cultural competence in nurses in clinical practice. Using the analyzed studies, we identified that attendance and distance training methods could impact the development of cultural competencies in nurses. Participants were offered several methods, such as face-to-face training, simulation training, eSimulation methods, and web-based learning.

The findings of this scoping review suggest that appropriate educational training can increase the cultural competence of nurses. These findings are supported by Cicolini et al. (2015), Yilmaz et al. (2017), Červený et al. (2020), and Antón-Solanas et al. (2021). Marja and Suvi (2021) report that simulations allow the integration of cultural elements into different vocational training and deepen the overall understanding of patient-centered cultural practices among simulation participants.

Workshops aimed at shaping culturally sensitive and competent attitudes, intensive and in-depth interactions with patients from different cultures, increasing knowledge of cultural issues, and intercultural communication training also strengthen the levels of cultural competence. There is a need to create smaller groups and increase practical hours to develop cultural competence (Majda et al., 2021).

Changing demographics make it necessary to prepare nurses to better meet the healthcare needs of patients from different cultural backgrounds. Cultural diversity in healthcare requires healthcare professionals to be aware of cultural needs and provide culturally appropriate healthcare (Turale et al., 2020). Cultural competence is essential in nursing since nurses spend more time in direct patient care than other medical staff (Gallagher and Polanin, 2015). Young and Guo (2016) report that cultural competencies develop through internal reflection and awareness over time. Findings of this review have shown that coping strategies are also an appropriate means of developing cultural competence.

According to Berlin et al. (2010), educational training should also include information on the cultural challenges and concerns of nurses and patients in the context of healthcare. Addressing these problems could improve daily clinical practice. Cultural competence in healthcare professionals improves patient satisfaction (Govere and Govere, 2016; Watt et al., 2016). Tosun (2021) add that integrating cultural education as an optional subject is insufficient because if nursing students did not choose the subject, they would not get the necessary information and skills to improve their culturally competent care.

This scoping review also shows the importance of overcoming language barriers and the role of interpreters in clinical practice. A systematic review by Govere and Govere (2016) recommends that training aimed at developing cultural competence includes the following topics or focuses: race, religion, sexual orientation, gender, and disability; vocal tones and nonverbal communication; and Latina Cultural Competence, Cultural Sensitivity program, Medical Spanish course, and Cultural Competencemodule.

Cai et al. (2021) draw attention to the need for practical cultural training. They note the need to identify and examine the factors that determine cultural competence. When offering training for cultural competence, there is often a risk of stereotyping since the training content often emphasizes minority groups and draws attention to the differences between minorities and the majority population. Such an approach should include a “do and do not” approach that defines how a nurse should treat a patient from each cultural background (Dogra, 2010).

### Limits of literary overview

This scoping review has several limits. The small number of studies analyzed is the main limitation of the study. Additionally, only studies available in English were included in the analysis. Moreover, studies from the “Grey literature” were not included, which may have led to the omission of some relevant studies.

## Conclusion

The increasing cultural diversity within global societies has created the need for cultural competence education in clinical practice nurses. The results of this scoping review point to possible methods for increasing cultural competencies among nurses. We report on several methods that can positively impact the development of cultural competence. Furthermore, the rapidly changing cultural demographics mean that societies need to constantly reassess the content of cultural diversity training so that participants are always prepared to provide culturally competent care. Cultural competence training greatly benefits nurses since it improves nurse–patient communication; however, it also benefits patients from different cultural backgrounds *via* improved healthcare and feelings of greater acceptance in society.

## Author contributions

MČ and VT: conception and design. MČ: data analysis and interpretation and manuscript draft. IK and VH: critical revision of the manuscript. MČ, IK, VH, and VT: final approval of the manuscript. All authors contributed to the article and approved the submitted version.

## Funding

This paper relates to the grant project 046/2021/S, supported by the Grant Agency of the University of South Bohemia in České Budějovice.

## Conflict of interest

The authors declare that the research was conducted in the absence of any commercial or financial relationships that could be construed as a potential conflict of interest.

## Publisher’s note

All claims expressed in this article are solely those of the authors and do not necessarily represent those of their affiliated organizations, or those of the publisher, the editors and the reviewers. Any product that may be evaluated in this article, or claim that may be made by its manufacturer, is not guaranteed or endorsed by the publisher.
